# Prehospital procedural sedation and analgesia agent selection: propofol, etomidate, ketamine

**DOI:** 10.1186/s13049-024-01218-w

**Published:** 2024-06-07

**Authors:** Sarper Yılmaz, Ali Cankut Tatlıparmak, Rohat Ak

**Affiliations:** 1 Dept. of Emergency Medicine, University of Health Sciences, Kartal Dr. Lutfi Kirdar City Hospital, Istanbul, Turkey; 2 Dept. of Emergency Medicine, Uskudar University Faculty of Medicine, Istanbul, Turkey

Dear Editor,

We have taken great interest in the study “Procedural sedation by advanced practice providers in the emergency medical service in the Netherlands: a retrospective study,” authored by Vliet and colleagues [1]. The research provides compelling data indicating that, particularly in recent years, there has been an increasing implementation of procedural sedation and analgesia (PSA).

Recent research has drawn attention to the utilization of analgesia, especially among trauma patients. It is imperative that the recognition and alleviation of pain be prioritized in the treatment of patients and the injured. Current pain management guidelines notably recommend the immediate application of procedural sedation and analgesia (PSA) at the point of triage [2]. Reflecting these guidelines, we have recently advocated for the inclusion of the START-A mnemonic into the Simple Triage and Rapid Treatment (START) protocol during mass casualty incidents to underscore the implementation of analgesia concurrently with triage. This proposal has garnered significant interest [3]. In disaster scenarios, where management of mass traumas is essential, early pain management similarly becomes a critical focus.

In Vliet and colleagues’ research, the predominant patient cohort subjected to PSA and sedation consisted of trauma patients in the pre-hospital phase. It is well-established that the vital parameters of trauma patients (blood pressure, pulse, respiratory parameters) serve as predictors that guide treatment approaches both pre-hospital and within hospital emergency departments, necessitating dynamic monitoring [4]. Recommendations advocate for the administration of ultra-short-acting agents, such as propofol or etomidate, in PSA for patients who are relatively healthy and hemodynamically stable [5, 6]. This study mainly utilized propofol in the chosen patient groups, with infrequent side effects. However, literature highlights a crucial consideration—the management of PSA and sedation in patients at risk of hypotension. Amid ongoing debates over blood pressure measurement and monitoring in the noisy, chaotic pre-hospital environment, the management of PSA or sedation agents that might influence a trauma patient’s blood pressure can add complexity to patient care [7].

In choosing agents for PSA and sedation in patients who carry a risk of hypotension—due to factors such as recent illness, dehydration, or heart disease—etomidate or ketamine is recommended. Both agents are preferred because they are known to maintain hemodynamic stability. In contrast, propofol is associated with a more substantial hypotensive effect [5]. While the hypotension induced by propofol is typically mild and transient, it can become significant in patients experiencing conditions such as trauma that might lead to hypovolemia and necessitate PSA [8].

Respiratory parameters, such as respiratory rate and blood oxygen saturation, which are critical pre-hospital vital parameters, require dynamic and meticulous management. In their study, Vliet and colleagues recommend using ketamine for PSA in patients who are at risk of airway or respiratory complications, particularly those who may present with potentially challenging airways or impaired respiratory functions. This recommendation comes despite propofol’s frequent use for PSA and sedation in their protocols [9].

In conclusion, the use of propofol for PSA and sedation in the pre-hospital phase, characterized by satisfactory outcomes and infrequent complications, might convey the image of it being the most reliable agent under the unique conditions of pre-hospital care. This perception can potentially foster misconceptions among clinicians and EMS personnel who administer these agents. There is an evident need for more extensive research into the choice of agents within this context. It is essential to acknowledge that the dynamics of injured patients’ vital parameters play a crucial role in their treatment; these parameters must be dynamically monitored and documented, and any administered agents, along with their administration times, must be rigorously evidenced and communicated to emergency medicine clinicians upon the patients’ arrival at emergency services.

## Data Availability

Not applicable.
